# Parenchyma Preserving Surgery for Idiopathic Chronic Calcific Pancreatitis in Children: A Report of Three Cases

**DOI:** 10.1089/pancan.2019.0008

**Published:** 2019-10-03

**Authors:** Tewodross Getu Wolde, Baobao Cai, Guo Feng, Junli Wu, Wentao Gao, Jishu Wei, Yi Miao

**Affiliations:** ^1^ Department of General Surgery, The First Affiliated Hospital of Nanjing Medical University, School of International Education NMU, Nanjing, People's Republic of China.; ^2^ Department of General Surgery, The Pancreas Center of Nanjing Medical University, The First Affiliated Hospital of Nanjing Medical University, Nanjing, People's Republic of China.

**Keywords:** Frey's procedure, idiopathic chronic calcific pancreatitis, modified Puestow procedure, pancreatic stones

## Abstract

**Background:** Idiopathic chronic calcific pancreatitis is a rare entity. Early surgical intervention and a parenchyma sparing procedure should be advocated to prevent further decay of the pancreas and the occurrence of cancer.

**Case Presentations:** Case 1: A 14-year-old boy presented with a 3-year history of right upper abdominal pain that has been aggravated in the last 2 months. Imaging revealed a dilated pancreatic duct of 6 mm with pancreatic duct stones in the head of pancreas. He underwent a Frey's procedure. Unfortunately, he was discharged with grade B pancreatic fistula. Case 2: A 12-year-old boy presented with a 1-year history of dull and recurring epigastric pain. Imaging studies showed multiple stones in a 12 mm dilated pancreatic duct. The patient underwent a modified Puestow procedure. Up to the 42th month follow-up, the patient had no pain complaints. Case 3: A 12-year-old boy with a 1-week history of a dull epigastric pain presented with with multiple stones in a 10 mm duct. He underwent a modified Puestow procedure and was discharged with alleviated pain.

**Conclusions:** “Conservative” surgery allows satisfactory pancreatic duct drainage, reduced rehospitalizations, and longer pain relief than alternative endoscopic procedures.

## Introduction

Chronic calcific pancreatitis (CCP) associated with pancreatic lithiasis is truly a rare finding in children. Few cases of children with pancreatic stones and chronic pancreatitis have been reported in the English literature. Our aim is to increase awareness of this diagnostically challenging disease, while laying emphasis on performing a parenchyma sparing surgery in children. We present three cases of idiopathic chronic calcific pancreatitis (ICCP) in three children aged 12, 12, and 14 years.

## Case 1

**FIG. 1.**
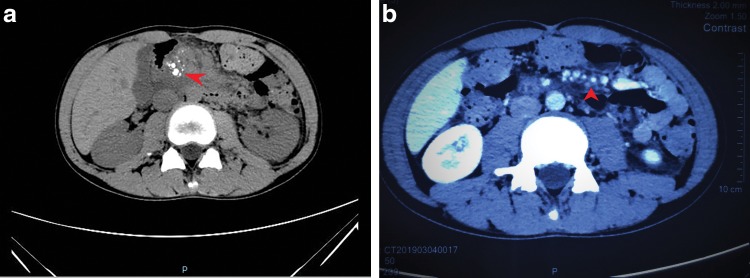
**(a, b)** Abdominal CT showing pancreatic duct stones in the body of pancreas with multiple high density calcifications in the head of pancreas. Red arrow: stones and pancreatic head calcification **(a)**; pancreatic duct stones **(b)**. CT, computed topography.

**FIG. 2.**
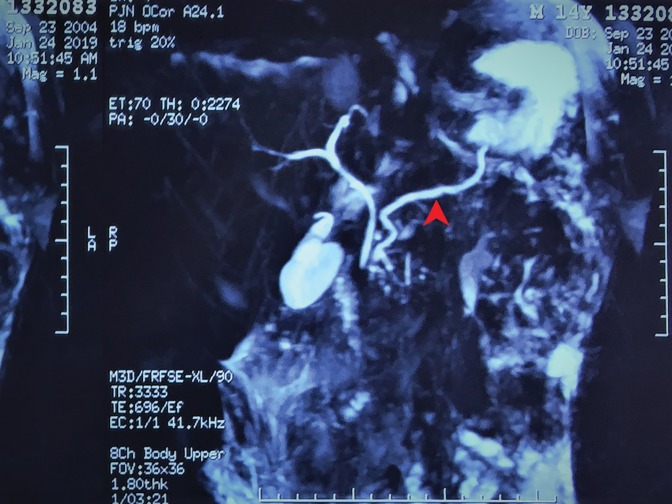
Magnetic resonance cholangiopancreatography showing a dilated main pancreatic duct of 6 mm (red arrow).

A 14-year-old boy presented to our center with a 3-year history of right upper abdominal discomfort accompanied by occasional painful episodes. The pain intensity worsened in January 2019 after the patient contracted a common cold. He underwent an abdominal computed topography (CT; Fig. 1a, b) that showed pancreatic duct stones in the body of pancreas and multiple high density calcifications in the head of pancreas; contour of the pancreas was unclear. A magnetic resonance imaging (MRI) indicated a dilated pancreatic duct containing multiple stones in the head pancreas with small amount of abdominal effusion. Magnetic resonance cholangiopancreatography (Fig. 2) showed a 6 mm dilated main pancreatic duct (MPD).

**FIG. 3.**
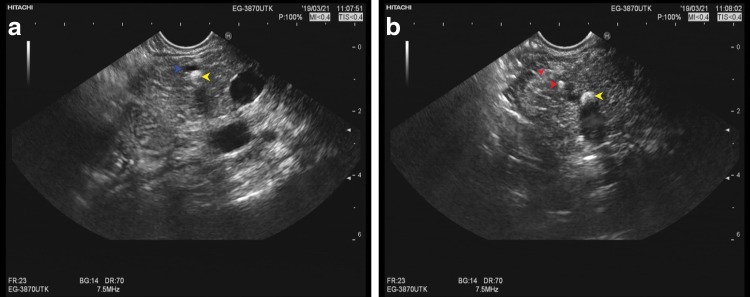
**(a, b)** Endoscopic ultrasound showing low pancreatic echoes, the internal echoes were uniform. Pancreatic duct in the head and neck of the pancreas showed multiple stone shadows (yellow arrow: pancreatic stones; red arrow: parenchyma calcification; and blue arrow: pancreatic duct).

Genes analysis on the 31st of January indicated a heterozygous mutation of lipase maturation factor 1 (LMF1; c1297G>A) gene (Table 1). According to the gene analysis report, this mutation is associated with a combined lipase deficiency. No family history of chronic pancreatitis was reported in first and second degree relatives. Physical examination and routine laboratory examinations did not reveal any positive findings. An increased level of tumor marker carbohydrate antigen 125 (CA-125; 109.4 U/mL) was noted. Test for immunoglobulin G4 was negative. An endoscopic ultrasound (EUS; Fig. 3a, b) revealed low pancreatic echoes with uniform internal echoes. The pancreatic duct showed multiple stone shadows in the areas of the head and neck of the pancreas.

**Table 1.** Summary of the Three Cases

	Case 1 (2019)	Case 2 (2015)	Case 3 (2009)
Time from onset of symptoms to definitive diagnosis	3 Years	1 Year	>7 Days
Symptom	Right upper abdominal pain and discomfort and painful episode	Dull epigastric pain and discomfort	Dull epigastric pain
BMI on admission:	184 cm, 70 kg	48 kg, 160 cm	
	BMI = 20.7 (66th percentile)	BMI = 18.7 (60th percentile)	
Tumor markers (CA-19-9 + CEA + CA-125 + CA-50)−	CA-125 increased (109.4 U/mL)	Normal CA-19-9, CA-125, CEA	Slightly increased CA-50 + CA-199 (26.9 and 43.94 KU/L, respectively)
Genetic analysis	Heterozygous mutation on LMF1 (c.1297G>A) gene. (LMF1; NM-022773.2; c.1297G>A; p.Asp433Asn; EX9; Het:missense mutation)	N/A	N/A
IgG4	(−)	(−)	(−)
PD dilation (mm)	6	12	10
Stone size in mm (intraoperative)	3–8	2–8	2–10
Management	Frey's procedure	Modified Puestow procedure	Modified Puestow procedure
Follow-up	2 Month follow-up: no abdominal pain or sign of pancreatic insufficiency	7th month: no pain	N/A
	Grade B pancreatic fistula	21st month: no pain	
		42nd month: no pain	

BMI, body mass index; CA, carbohydrate antigen; CEA, carcinoembryonic antigen; IgG4, immunoglobulin G4; LMF1, lipase maturation factor 1; N/A, not available; PD, pancreatic duct.

**FIG. 4.**
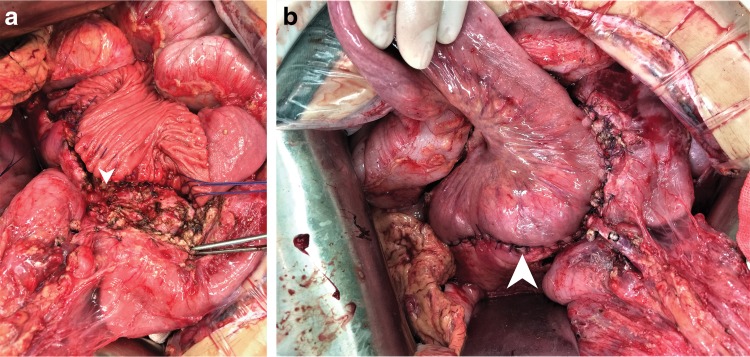
**(a)** The cored out pancreatic head and distal pancreatic dichotomy in the Frey procedure. **(b)** Reconstruction of the single side to side Roux-en-Y pancreaticojejunostomy. White arrow: cored out pancreatic head **(a)**; Roux-en-Y pancreaticojejunostomy **(b)**.

An EUS guided fine-needle aspiration cytology (FNAC; March 19, 2019) did not reveal any special finding. Nine days after EUS the patient complained of chest tightness and discomfort. Chest X-ray and CT showed a large amount of pleural effusion on the left lung and right deviation of mediastinum. This pancreatic pleural effusion potentially occurred due to duct injury during the EUS-FNAC procedure. Consequently, a closed thoracic drainage was performed with removal of 600 mL on the 1st day and <1000 mL on the following days. On April 9, 2019, the patient underwent a Frey's procedure (lateral [longitudinal] pancreaticojejunostomy or LPJ; Fig. 4a, b).

Intraoperatively a high pressure flow of pancreatic juice was observed after stone removal and an obvious dilation of the pancreatic duct was noted. Multiple white stones ranging from 0.3 to 0.8 cm were retrieved. Rapid intraoperative frozen section of inflamed pancreatic tissue and pancreatic duct did not reveal any malignant changes. On postoperative day 13 the patient was discharged with alleviated symptoms. Unfortunately, he developed a grade B pancreatic fistula (International Study Group of Pancreatic Fistula 2005).

## Case 2

In October 2015, a 12-year-old boy presented to our center with a 1-year history of dull recurring epigastric pain and discomfort. The patient has previously been treated with acid suppressing agents in a primary care center. However, only temporary pain relief was achieved and the pain gradually started to worsen. No history of associated back pain or vomiting has been reported. Additionally no history of drug, alcohol intake, or trauma was noted. An MRI report on September 2015 revealed multiple pancreatic duct stones.

The patient was then admitted to our center for further examination and treatment. The patient had no family history of chronic pancreatitis. No special findings were observed upon physical examination. Laboratory examination indicated a low blood glucose (lowest 3.62 mmol/L) and low creatinine (lowest 35.7 μmol/L) level, elevated alkaline phosphatase (ALP; 272.8 U/L), and normal alpha fetoprotein, carcinoembryonic antigen, CA-19-9 level (1.8 ng/mL, 0.2 ng/mL, and 7.6 U/mL, respectively). On the day of admission the patient underwent an abdominal CT that showed unclear gland contour with multiple calcification and small pseudocyst; these features were suggestive of chronic pancreatitis. Multiple stones in a 12 mm dilated pancreatic duct were also noted.

EUS showed a gray–red irregular structure of 0.3 cm diameter on the pancreatic duct wall. Pathological result obtained through EUS-FNA of the pancreatic duct wall suggested the presence of few extruded and deformed glandular duct-like structures with mild atypia of the glandular duct. On the 14th of October the patient received a modified Puestow procedure (side-to-side LPJ). Intraoperatively, an atrophied parenchyma with a dilated and high pressure MPD were observed containing multiple white, staghorn-shaped stones ranging from 0.2 to 0.8 cm in diameter in the region of the head and uncinate process.

**FIG. 5.**
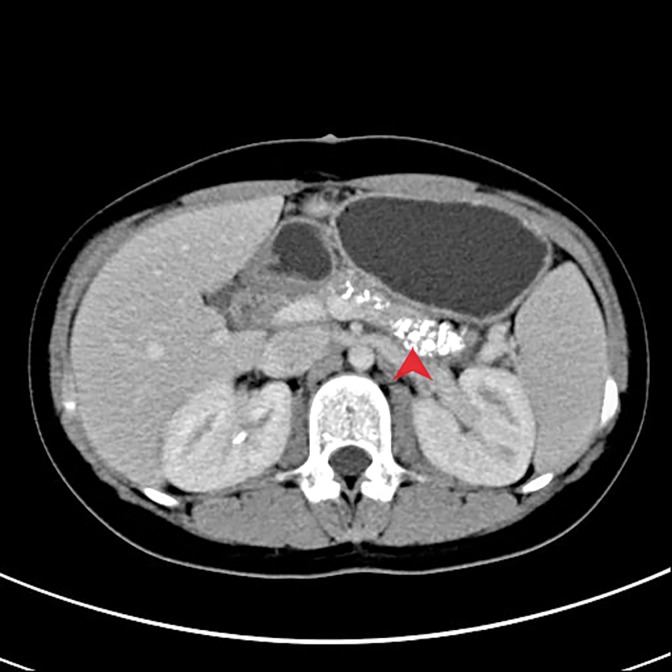
Seventh month follow-up. Abdominal CT showing parenchyma calcification. Red arrow: pancreatic calcification.

Rapid intraoperative frozen section of the pancreatic duct wall specimen showed few heteromorphic glands in fibrous tissue. Postoperative pathological studies did not indicate any sign of malignancy. The patient did not have any postoperative complication and was discharged on postoperative day 7 with an uneventful recovery. On the 7th month follow-up (May 23, 2016), abdominal CT (Fig. 5) revealed chronic pancreatitis. Nonetheless, there was no pancreatic duct dilation and the patient remained pain free. Despite having no pain on the 21st month follow-up, abdominal CT indicated chronic pancreatitis with dilation of pancreatic duct in contrast to the 7th month abdominal CT. On the 42th month follow-up the patient was still pain free.

## Case 3

On March 17, 2009, a 12-year-old boy was admitted to our center for a dull epigastric pain that lasted more than 7 days before admission. There was no history of associated vomiting or radiating back pain. No hazardous drug intake or trauma was reported. Previous abdominal CT in Shihong County Hospital (March 11, 2009) indicated chronic pancreatitis with an extensively calcified parenchyma of the pancreas. No familial history of chronic pancreatitis was noted. Physical examination revealed reticular skin ecchymosis around the umbilicus with a diameter of 6–8 cm. Laboratory examinations showed increased ALP level (324.8 U/L), increased glucose level (7.66 mmol/L), and slightly increased CA-50 and CA-199 (26.9 and 43.94 KU/L, respectively).

A modified Puestow procedure was carried out (March 27, 2009). The pancreatic duct had a 10 mm diameter with multiple white sand-like stones ranging from 0.2 to 1.0 cm. An atrophied pancreatic parenchyma was observed. Postoperative pathology reports did not indicate any sign of malignancy. The patient did not suffer from any postoperative complications and was discharged on postoperative day 11 with improved symptoms.

## Discussion

In adult chronic pancreatitis the main etiological factors include alcohol, smoking, and environmental toxins.^1,2^ However, the causes remain more elusive in children. The widely used TIGAR-Ot risk factor classification system details the risk factors rather than the direct causes of chronic pancreatitis. They are classified as toxic-metabolic (alcohol medication, etc.); idiopathic, genetic; autoimmune; recurrent and severe acute pancreatitis; and obstructive (pancreatic divisum, etc.).^3^

Hereditary pancreatitis can be defined as a patient with recurrent acute or chronic pancreatitis who has a first or second degree relative having similar presentation or one who presents with mutated SPINK1, PRSS1, CFTR, and CTRC genes. While an autosomal dominant inheritance pattern is the most common form of inheritance, another method of inheritance is in an autosomal recessive fashion whereby 2 mutated parental genes are needed for the phenotypic manifestation (e.g., CFTR gene and SPINK1).^4^ The autosomal recessive inheritance is a perplex pattern requiring environmental factors and genetic mutation or a mix of individual mutated genes.^4^ In the absence of known mutations, trauma, alcohol consumption, drug intake, infection, or metabolic disorders, it can be referred as idiopathic chronic pancreatitis.^5^

Genetic analysis case 1 revealed a heterozygous mutation on the LMF1 gene. In a research article by Péterfy et al.,^6^ homozygous mutation of the LMF1 has been suggested as potential candidate gene in hypertriglyceridemia and may be associated with recurrent attacks of pancreatitis. However, the exact role of this mutation in this case is unclear since the patients has normal triglyceride levels (0.62 mmol/L), normal lipoprotein-A (165 mg/mL), normal total cholesterol (2.51 mmol/L), low-density lipoprotein (LDL) (1.55 mmol/L), and decreased LDL (1.55 mmol/L). To the best of our knowledge LMF1 gene mutation has not yet been added to the pool of culprit genes of hereditary pancreatitis. Although gene analysis was not available for cases 1 and 2, these patients did not have any first degree or second degree relative with a history of pancreatitis.

ICCP is characterized by chronic inflammation with calcification of the parenchyma with pancreatic duct lithurgia.^7^ Calcification of parenchyma is the result of repetitive bouts of pancreatitis. These recurrent attacks lead to deposition of proteinaceous plugs in pancreatic ducts that eventually accumulate calcium carbonate and lead to calcification. The disruption of pancreatic duct patency leads to recurrent abdominal pain.^7^ The diagnosis of CCP in children remains a challenging task for the clinician due to the rarity of the condition in the pediatric group. As demonstrated in the above three cases the time from initial symptom presentation to definitive diagnosis ranged 1 week to 3 years.

The objective when treating CCP in children is pain alleviation, correction of any endocrine/exocrine insufficiency, and improving the drainage system of the pancreas.^8^ Despite noninvasive procedures such as sphincterectomy and endoscopic retrograde cholangiopancreatography being achievable, these do not address the fundamental problem of drainage insufficiency in the pancreas therefore requiring multiple procedures and multiple rehospitalizations. Some studies have shown very good results regarding the safety and efficacy of endotherapy in a select group of patients.^9,10^

However, multiple studies have demonstrated early surgical management to be superior in term of pain relief and improved quality of life.^11,12^ A prospective study by Cahen et al.^11^ showed that even after 6 years, patients who first received surgical treatment had improved pain relief (80% were pain free against 38% in the endoscopy group) and had fewer procedures than the endoscopic group. Early surgical intervention had a better outcome than endoscopic ones (including extracorporeal shockwave lithotripsy)^11^ It is of major importance to make surgery the earliest treatment modality in painful chronic pancreatitis pediatric patients due to the potential of lower risk of pancreatic insufficiency and reintervention.^12,13^

Furthermore, a very interesting trial is underway: the early surgery versus optimal current step-up practice for chronic pancreatitis (ESCAPE) trial from the Dutch Pancreatitis Study Group. Early reports presented at the United European Gastroenterology Week 2018 have confirmed that early surgical treatment was superior to the step-up approach.^14^ Therefore, the only definitive management should be early surgical management (<3 years from diagnosis). Laje and Adzick^13^ reported satisfactory results with LPJ for pain relief in obstructive chronic pancreatitis. Similarly, Hodgman et al.^15^ reported the largest single center experience with modified Puestow procedure on children with chronic pancreatitis. The majorities of patients had a significant pain improvement or were pain free after the procedure.^15^

More radical surgeries such as total pancreatectomy and islet cell transplantation are possible but do not seem to offer any additional benefits but instead may increase the probability of inducing insulin-dependent diabetes.^16^ Reports from Bellin et al.^17^ have shown only 56% of children who underwent total pancreatectomy and islet-transplantation were not dependent on insulin and an astonishing 39% needed narcotics for pain relief. Concurrently, another series by Chinnakotla et al.^18^ showed a 41.3% achieved insulin independency.

LPJ and Frey's procedure are the modalities of choice in our center because these procedures allow the pancreas to be adequately drained meanwhile maximizing preservation of pancreatic parenchyma, thereby reducing any endocrine/exocrine insufficiency seen with more radical operations. The Frey's procedure has potential benefits as it is a “head coring” technique in which fibrotic tissue from the pancreatic head is removed *en bloc* and allows for a better duct drainage than the modified Puestow procedure. This fibrotic area is thought to play a central role in pain in this condition.^19^ Therefore, a Frey's procedure should have a potentially lower pain recurrence and a better duct drainage than the modified Puestow procedure.

Moreover, it is known and well-documented that the chronic inflammatory process in chronic pancreatitis may induce the eventual progression to pancreatic cancer.^20^ Early surgery for chronic pancreatitis significantly reduces and may even halt the evolution to cancer.^21^ Therefore, parenchyma preserving surgery not only reduces pain recurrence and pancreatic insufficiency but may also decrease the risk of developing cancer in the years ahead.

## Conclusions

ICCP is a rare finding in children with only three cases in our center from 2008 to April 2019. Parenchyma-sparing surgery for children with ICCP is highly beneficial because it preserves a maximum of pancreatic tissue thereby potentially reducing any exocrine and endocrine deficiencies associated with more radical procedures. Moreover, early surgery allows satisfactory pancreatic duct drainage and reduced rehospitalizations and higher pain relief than alterative endoscopic procedures.

## Abbreviations Used

ALP: alkaline phosphatase
BMI: body mass index
CA-125: carbohydrate antigen 125
CCP: chronic calcific pancreatitis
CEA: carcinoembryonic antigen
CT: computed topography
EUS: endoscopic ultrasound
FNAC: fine-needle aspiration cytology
ICCP: idiopathic chronic calcific pancreatitis
IgG4: immunoglobulin G4
LMF1: lipase maturation factor 1
LPJ: lateral [longitudinal] pancreaticojejunostomy
MPD: main pancreatic duct
MRI: magnetic resonance imaging
